# Kelvin probe force microscopy in liquid using electrochemical force microscopy

**DOI:** 10.3762/bjnano.6.19

**Published:** 2015-01-19

**Authors:** Liam Collins, Stephen Jesse, Jason I Kilpatrick, Alexander Tselev, M Baris Okatan, Sergei V Kalinin, Brian J Rodriguez

**Affiliations:** 1 School of Physics, University College Dublin, Belfield, Dublin 4, Ireland; 2 Conway Institute of Biomolecular and Biomedical Research, University College Dublin, Belfield, Dublin 4, Ireland; 3 Center for Nanophase Materials Sciences, Oak Ridge National Laboratory, Oak Ridge, Tennessee 37831, USA; 4 Institute for Functional Imaging of Materials, Oak Ridge National Laboratory, Oak Ridge, Tennessee 37831, USA

**Keywords:** diffuse charge dynamics, double layer charging, electrochemical force microscopy, electrochemistry, Kelvin probe force microscopy

## Abstract

Conventional closed loop-Kelvin probe force microscopy (KPFM) has emerged as a powerful technique for probing electric and transport phenomena at the solid–gas interface. The extension of KPFM capabilities to probe electrostatic and electrochemical phenomena at the solid–liquid interface is of interest for a broad range of applications from energy storage to biological systems. However, the operation of KPFM implicitly relies on the presence of a linear lossless dielectric in the probe–sample gap, a condition which is violated for ionically-active liquids (e.g., when diffuse charge dynamics are present). Here, electrostatic and electrochemical measurements are demonstrated in ionically-active (polar isopropanol, milli-Q water and aqueous NaCl) and ionically-inactive (non-polar decane) liquids by electrochemical force microscopy (EcFM), a multidimensional (i.e., bias- and time-resolved) spectroscopy method. In the absence of mobile charges (ambient and non-polar liquids), KPFM and EcFM are both feasible, yielding comparable contact potential difference (CPD) values. In ionically-active liquids, KPFM is not possible and EcFM can be used to measure the dynamic CPD and a rich spectrum of information pertaining to charge screening, ion diffusion, and electrochemical processes (e.g., Faradaic reactions). EcFM measurements conducted in isopropanol and milli-Q water over Au and highly ordered pyrolytic graphite electrodes demonstrate both sample- and solvent-dependent features. Finally, the feasibility of using EcFM as a local force-based mapping technique of material-dependent electrostatic and electrochemical response is investigated. The resultant high dimensional dataset is visualized using a purely statistical approach that does not require a priori physical models, allowing for qualitative mapping of electrostatic and electrochemical material properties at the solid–liquid interface.

## Introduction

Many important physical, chemical and biological processes including wetting, adsorption, electronic transfer and catalysis take place at the solid–liquid interface [1–2]. Very often these processes involve charge storage through the formation of electric double layers adjacent to an electrode surface (i.e., capacitive storage) and/or transfer of electrons across an electrode–electrolyte interface (i.e., pseudocapacitive storage). Consequently, understanding the local electrostatic, electrochemical and double layer ion dynamics at the solid–liquid interface is crucial to the study of corrosion, sensing, energy storage and bioelectric interfaces [3]. These processes are dynamic in nature, involving changes of the local concentration of ions through migration (field-driven ion transport) and diffusion (concentration-gradient-driven transport) both to and from the solid–liquid interface as well as electron transfer reactions across the interface, resulting in a broad spectrum of charge relaxation timescales (ns–s) [4–10].

In electrochemical systems, the local reactivities and overpotentials of nucleation centers across micro- to nanometer length scales ultimately govern the electrochemical functionality, lifetime and failure mechanisms of materials and devices. Understanding such systems necessitates the development of characterization techniques capable of operating in ionically-active liquids across multiple length scales from a single step edge or point defect up to the device level. While macroscopic electrochemical measurements are capable of probing material properties on the device level, few techniques are capable of operating below the micron length scale [11]. Scanning probe microscopy (SPM) techniques are uniquely positioned to probe structure on nano- to micrometer length scales and can do so under vacuum, ambient or liquid environments. Thus, the development of SPM techniques that are capable of obtaining both structural information and information on local electrochemical functionality is a natural combination of capability and necessity [12–15].

In the past few decades, a plethora of SPM techniques capable of probing electrostatic, [16–17] electromechanical [18], electrochemical [19] and ionic [15] functionality on the nanoscale have been developed. A paradigmatic example of such development is closed loop-Kelvin probe force microscopy (KPFM) [20], which has become a widely used voltage-modulated SPM technique for the measurement of surface potential distribution, and has proven to be an important technique for studying electronic functionality at the solid–gas interface. KPFM measurements have previously been utilized to investigate surface photo-voltage in photovoltaics [21–23] and charging dynamics in ferroelectric [24–26], dielectric [27] and ionic materials [14,28–29]. When operated in ultra-high vacuum, KPFM has been demonstrated to provide absolute surface potential measurements, with molecular and atomic scale resolution previously reported [30–32]. Interpretation of surface potential values from this technique can, however, become complicated by feedback artefacts and stray capacitance even in vacuum [33–35]. In ambient environments, the interpretation of surface potential values increases in complexity due to the possible shielding of the surface by mobile adsorbates and the presence of a thin water layer, resulting in an unknown background potential [36].

The study of, e.g., biological systems and battery materials necessitates the application of KPFM-like techniques in ionically-active liquids whilst presenting an opportunity to overcome the difficulties present under ambient conditions. Despite the urgent need for KPFM-like measurements in ionically-active liquids, suitable techniques have yet to be established due to complications arising from the broad spectrum of relaxation timescales associated with diffuse charge dynamics [37–38].

Previous attempts to implement KPFM in liquid aimed to avoid ion dynamics and electrochemical processes [39–43]. One approach to avoid diffuse charge dynamics has been to implement KPFM in non-polar solutions [39]. Open loop-KPFM approaches, offering a promising approach for measuring electrostatic properties in ionically-active liquids, have also been previously reported [40–43]. In general, open loop-KPFM does not require the application of a DC bias via a feedback loop and can be performed by utilizing either (i) both AC voltage and DC bias (referred to here as open loop bias spectroscopy, OLBS) [44], or (ii) AC voltage alone (referred to here as dual harmonic KPFM, DH-KPFM) [34,45]. Kobayashi et al. [40–42] have previously demonstrated the application of DH-KPFM for surface potential mapping in low molarity solutions (<10 mM). However, for energy or biological applications, where solution concentrations are often >>10 mM, ion dynamics occur at timescales <<100 ns [43]. The broad distribution of timescales associated with electrochemical processes necessitates the development of techniques capable of probing ion dynamics and electrochemical processes taking place between the probe and sample as a function of time.

Here, OLBS measurements in air and milli-Q water are compared to illustrate the infeasibility of implementing KPFM in ionically-active liquids. A multidimensional (i.e., bias- and time-resolved) spectroscopy method, referred to as electrochemical force microscopy (EcFM) [38], is subsequently investigated for performing electrostatic measurements in both ionically-active and -inactive liquids. EcFM is employed to detect the bias- and time-dependent electrostatic and electrochemical forces between probe and sample under ambient, non-polar (ionically-inactive decane) and polar (ionically-active isopropanol, milli-Q water and aqueous NaCl) environments. The measurement of bias- and time-dependent ion dynamics allows different electrokinetic phenomena to be separated and a set of environmental and measurement timescale requirements for determining CPD under conditions comparable with KPFM to be delineated. Finally, the applicability of EcFM to resolve local electrostatic and electrochemical properties at the solid–liquid interface in the presence of diffuse ion dynamics is demonstrated. The increase in both size and complexity of the resulting data necessitates simple analysis methods capable of dealing with high dimensional information without relying on a priori physical models. Multivariate statistical approaches are implemented in order to map the local electrochemical behavior across a metal-insulator junction.

## Results and Discussion

### OLBS in air and milli-Q water

Assuming a lossless dielectric between probe and sample, the static and dynamic components of the electrostatic force resulting when a DC bias, *V*_dc_, and AC voltage, *V*_ac_, at a frequency, ω, is applied between a conducting probe and a sample (*V*_probe_ = *V*_dc_ + *V*_ac_ cos(ω*t*)) are given by:

[1]
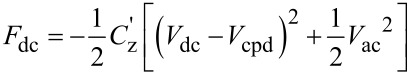


[2]
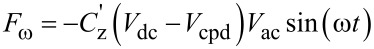


[3]
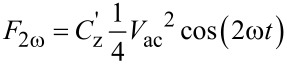


where 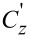 is the capacitance gradient, governed by the geometry and permittivity of the probe–sample capacitor system and *V*_cpd_ is the CPD or electrochemical potential between the probe and sample. Equation 2 and Equation 3 describe the bias-dependent first harmonic force, *F*_ω_, and the bias-independent second harmonic force, *F*_2ω_, acting on the probe, which are detected as the first and second harmonic cantilever amplitude (*A*_ω_ and *A*_2ω_) and phase (θ_ω_ and θ_2ω_) using lock-in techniques. Equation 2 predicts a linear dependence of *F*_ω_ with respect to the probe–sample DC bias, which is minimized when *V*_dc_ = *V*_cpd_. KPFM employs this principle via a feedback loop to minimize *A*_ω_. Depending on the material under investigation and the environment in which the measurement is performed, the CPD can then be related to various electrostatic and electrochemical properties. For example, in the case of a metal probe with known work function in vacuum, the CPD provides a measurement of the work function of the sample. For non-conducting materials, such as semiconductors, dielectrics and ferroelectrics, additional Columbic terms arising from static charges and polarizability may also contribute to the detected force [46]. *F*_2ω_ can be used to study local dielectric properties [47–48], and has recently been used to quantify dielectric constants in low molarity (<10 mM) solutions at high frequency (>MHz) [49–50].

In deriving Equation 2, an implicit assumption of a linear lossless dielectric between probe and sample is made. This has two major implications, which underpin the operation of KPFM. First, the electrostatic force must be measured under equilibrium conditions and secondly, *F*_ω_ must have a linear *V*_dc_ dependence. In ionically-active liquids, however, where a lossy polarizable dielectric is present between probe and sample, Equation 2 is insufficient to accurately describe the system. In particular, if the measurement timescale >> relaxation time of the system, the system will be in equilibrium with the double layer fully formed, whereas for a measurement timescale << the relaxation time of the system, the system will behave similar to a linear dielectric. Finally, intermediate relaxation times can give rise to complex dynamic responses. Hence, bias-induced charge dynamics (e.g., electromigration and ion diffusion) as well as steric effects [6–9] and electrochemical processes at larger biases, need to be detected and separated experimentally in order to fully characterize the system, which is beyond the capabilities of KPFM.

**Figure 1:**
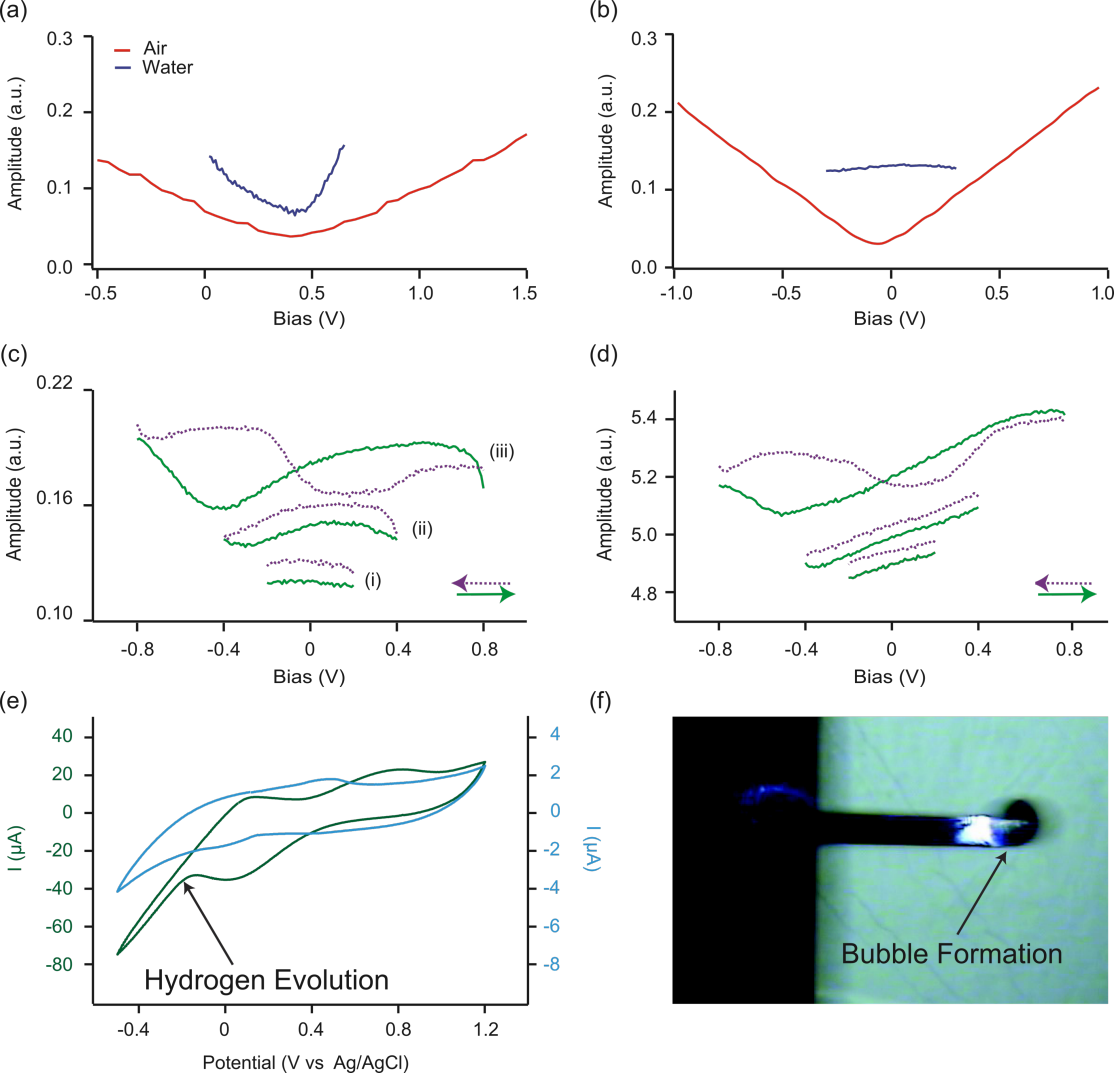
*A*_ω_ recorded in air (red) and milli-Q water (blue) 50 nm above (a) HOPG and (b) Au surfaces as a function of the DC bias applied to the probe. (c) *A*_ω_ and (d) *A*_2ω_ recorded 50 nm above an Au surface in the order of (i) small (±200 mV), (ii) medium (±400 mV) and (iii) large (±800 mV) bias ranges. Measurements were performed using a sweep rate of 500 mVs^−1^ for HOPG and 100 mVs^−1^ for Au in air and 100 mVs^−1^ for both surfaces in milli-Q water, with *V*_ac_ = 0.5 V [5 kHz] applied to the probe. (e) Cyclic voltammetry measurements in milli-Q water of HOPG (blue) and Au (green) electrodes were performed using a sweep rate of 50 mVs^−1^. (f) Optical image of bubble formation between the probe and HOPG surface in response to the application of a 2 V DC bias (nominal cantilever length is 225 µm).

The bias dependence of *A*_ω_ and *A*_2ω_ recorded during OLBS is shown in Figure 1. Here, *V*_ac_ was applied to the probe at a fixed distance (200 nm) above the surface as *V*_dc_ was ramped linearly to a set potential, at which point the *V*_dc_ ramp was inverted. Measurements were performed in air and milli-Q water with the same Pt/Ir coated probe. Figure 1a,b shows the bias dependence of *A*_ω_ for highly ordered pyrolytic graphite (HOPG) and Au surfaces, respectively. For both HOPG and Au, a linear dependence of *A*_ω_ to the applied *V*_dc_ was observed in air, and the minimum of *A*_ω_ corresponds to the measured CPD of the probe–sample system, as described by Equation 2. Thus, the CPD was estimated to be ≈420 mV for HOPG and ≈−60 mV for Au.

To investigate the operation of KPFM in liquid, the measurements were repeated in milli-Q water, which has a minimum ion concentration of ≈4 × 10^−7^ M [51]. In a first set of experiments, small bias sweeps (±300 mV) were performed to reduce the likelihood of inducing irreversible electrochemical processes. For HOPG, a minimum was observed at ≈370 mV, close to the measured CPD in air, but no minimum was observed for Au. The observed *A*_ω_ for Au is weakly dependent on bias in this range, suggesting the mechanisms at play are significantly different from those described by Equation 2. In aqueous solutions, charge screening from ions, adsorption of water molecules and surface dipoles at the probe and/or sample surface could also play a role in shifting the measured CPD of the Au surface outside of the bias range studied.

In order to investigate the possibility that the CPD of Au was shifted in liquid, and to further investigate the dependence of electrostatic forces at larger biases, *A*_ω_ and *A*_2ω_ were probed under increasing bias ranges, i.e., adopting a first order reversal curve approach. Previously, this approach was demonstrated to be highly effective in exploring local bias-induced phenomena that are reversible for small biases and irreversible at high biases [14,52–53].

Typical results for *A*_ω_ and *A*_2ω_ above an Au surface for three bias ranges are shown in Figure 1c,d. Data sweeps were collected for a small (±200 mV), medium (±400 mV) and large (±800 mV) bias range, consecutively. For small bias sweeps (±200 mV), finite shifts in the magnitude of *A*_ω_ between negative and positive (green) and between positive and negative (purple) bias sweeps were observed, likely resulting from a redistribution of ions. For medium bias sweeps (±400 mV), hysteretic behavior was observed between sweeps. Large bias sweeps (±800 mV) resulted in complex responses including hysteretic behavior and the presence of maxima and minima. Raiteri et al. reported similar hysteretic behavior in the static electrochemical stress experienced for biased Au electrodes in a variety of electrolytes [54]. Umeda et al. also observed similar hysteresis in this bias range for a cantilever above a platinum surface in water [55]. For all bias sweeps shown here, the general shapes of the OLBS curves were reproducible when using the same sweep rate; however, the shape and magnitude of the response was found to depend heavily on sweep rate (not shown), suggesting an underlying temporal dependence of the response.

The differences in the bias-dependent *A*_ω_ response in milli-Q water and air for HOPG and Au are attributed to the differences in electronic and electrochemical properties of the materials. This was confirmed macroscopically using cyclic voltammetry (CV) measurements [56]. Typical CV traces for both HOPG and Au electrodes in milli-Q water are shown in Figure 1e. In some respects, OLBS (Figure 1a–d) is a force-based analogue of the macroscopic current-based CV measurement.

Hydrogen evolution can be observed for Au at potentials of <−200 mV (Figure 1e), but not for HOPG, showing that HOPG is more electrocatalytically inert than the Au electrode. In OLBS measurements, when using bias sweeps larger than 2 V, large changes in the AFM cantilever deflection signal occurred (not shown), often followed by visible bubble nucleation in the probe–sample gap (e.g., Figure 1f). Attempts at implementing KPFM in ionically-active liquids will often result in similar, if not more catastrophic, bubble formation by virtue of the absence of a defined minimum, which may result in the application of large DC biases by the feedback loop.

Figure 1 demonstrates that the universal application of KPFM across all materials, all bias ranges and all solutions is not viable. In particular, the absence of a unique minimum in *A*_ω_ and the presence of hysteresis and irreversible reactions at larger biases observed for Au (Figure 1c,d) are fundamental barriers to the proper application of KPFM in milli-Q water and other ionically-active liquids.

### Bias- and time-resolved EcFM

The hysteresis observed in Figure 1 illustrates that the response in milli-Q water is more complex than expected from the electrostatic force interactions described by Equations 1–3. This is not surprising as the underlying assumption of a lossless dielectric is no longer valid in the presence of diffuse ion dynamics, precluding the use of KPFM. The observed hysteretic response can be explained as a combination of tip–sample interactions caused by field-driven migration and concentration-gradient-driven diffusion of ions in the bulk electrolyte, as well as possible steric effects, double layer charging and electrochemical processes at larger biases. This necessitates making simultaneous measurements as a function of both bias and time. As a relevant parallel, macroscopic analysis of diffuse charge dynamics or electrochemical processes also requires separation of the electrokinetic effects, which cannot be obtained using linear bias sweeps (e.g., CV measurements), ultimately requiring either pulsed electrochemical or impedance measurements [57].

**Figure 2:**
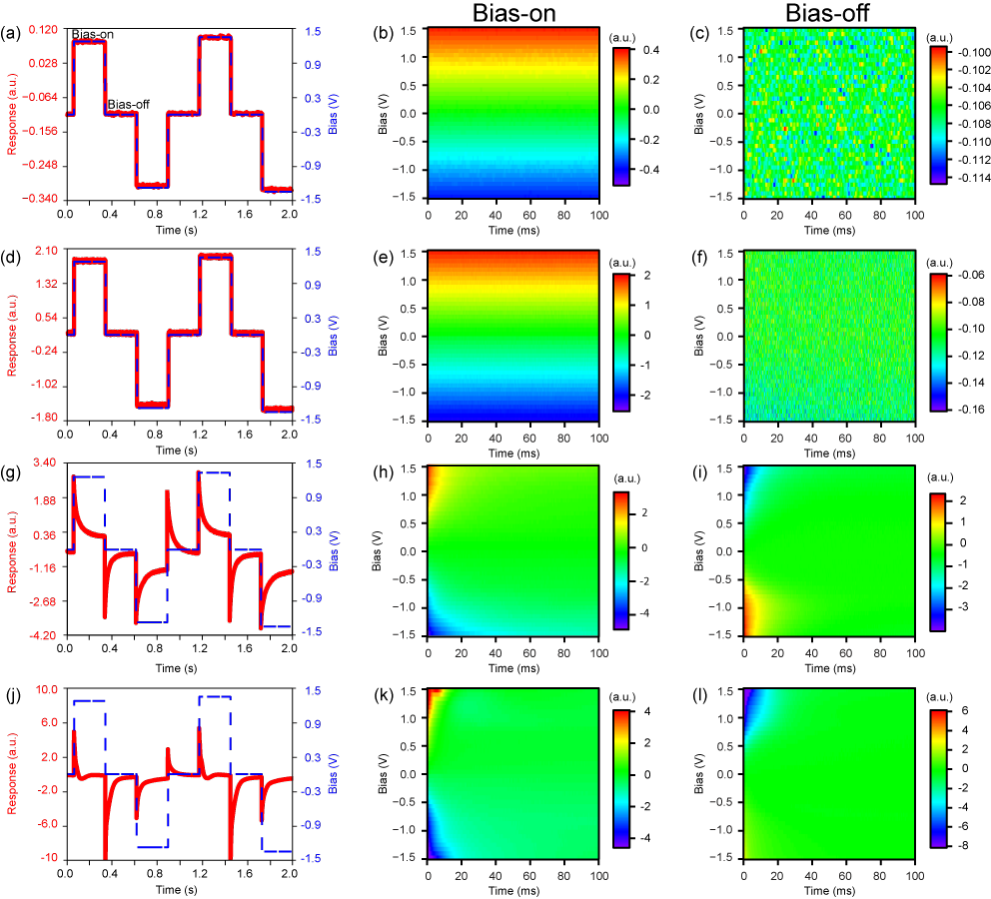
EcFM 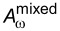 response collected 200 nm above a grounded Au electrode in (a, b, c) air, (d, e, f) decane and (g, h, i) isopropanol and (j, k, l) milli-Q water. (a, d, g, j) Temporal response (solid red line) of 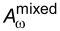 in response to the applied DC bias waveform (dashed blue line). Single-point EcFM 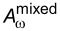 spectra showing bias-on (b, e, h, k) and bias-off (c, f, i, l) states. Measurements were performed with *V*_ac_ = 0.5 V [25 kHz] applied to the probe.

To achieve this goal, a multidimensional spectroscopic strategy was implemented, which is capable of probing both the bias- and time-dependent dynamic probe-sample interaction, referred to as EcFM [38]. In EcFM the probe is electrically modulated with a high frequency AC voltage used to detect the dynamic cantilever response using lock-in amplifiers while the system is perturbed by DC bias waveforms applied, in the present study, to the probe. Ideally, the AC voltage excitation should be at sufficiently high frequency such that AC voltage-induced electrochemical processes do not dominate the response mechanism and thus the system is probed under quasistatic conditions. In EcFM, the data is collected both during the bias application (bias-on state) and following the bias application (bias-off state), as the magnitude of the bias pulses is increased linearly with time. Figure 2a shows a section of the DC bias waveform applied to the probe with the corresponding response recorded in air. Here, data is presented as the mixed response (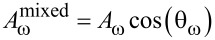) which contains information on the polarity and magnitude of the signal. Little to no relaxation of the electrostatic force is observed, with 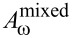 following the applied bias, and therefore satisfying the second principle of KPFM, a time-invariant electrostatic response. This is expected for a purely electrostatic response, or more generally when the force experienced by the system is governed purely by the time-independent Maxwell stress tensor directly related to the charge density between probe and sample [58].

The data can be presented as an EcFM spectra representing the bias- and time-dependent 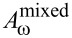 for a single location, e.g., Figure 2b. In ionically-inactive non-polar liquids, such as decane, Figure 2d,e,f, a similar response is observed to that observed in air, where again the electrostatic force follows the applied DC bias and is time-invariant. In ionically-active polar liquids, such as isopropanol (Figure 2g,h,i) or milli-Q water (Figure 2j,k,l), a very different response mechanism is observed. For these systems, a large increase in 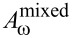 is observed at the instant the bias pulse is applied, which relaxes within ≈10 ms to below ≈30% of the peak value. In isopropanol, a smooth transient decay of the response is observed for all biases measured. The relaxation could be well-described by a double exponent decay having a fast relaxation time (τ_1_) of 1.2 ms to 6.3 ms and a slower relaxation time (τ_2_) between 11 ms and 47 ms (see Supporting Information File 1, Figure S1 for full fitting results). For milli-Q water, regions of varying transient response could be identified. For bias values between –1.5 V and 0.6 V a smooth transient decay having a τ_1_ of 4.4 ms and a slower relaxation τ_2_ between 12 ms and 50 ms were determined (see Supporting Information File 1, Figure S2 for full fitting results). In milli-Q water for larger positive biases (>600 mV), a more complex response with local minima were observed (>3 ms), as seen in Figure 2j, which could not be described by exponential fitting alone and are likely indicative of the initiation of Faradaic processes between tip and sample.

Similar information can be obtained from the bias-off state. For both air and decane the bias-off state was time-invariant for the entire bias range, Figure 2c,f. For isopropanol (Figure 2i) and milli-Q water (Figure 2l), transient responses were detected for the bias-off state and the magnitude of the response was bias-dependent. This response is attributed to the redistribution of ions in the double layer that occurs when the bias is switched off. For milli-Q water (Figure 2l), the largest response (including for bias-on states) was seen after the application of positive bias pulses greater than +600 mV. Interestingly, this is also the region where deviations from an exponential decay in the bias-on state are detected, which may be indicative of, e.g., steric effects or Faradaic reactions being induced at large positive biases. EcFM measurements in a three electrode configuration with a reference and counter electrode would be beneficial for elucidating the precise bias at which electron transfer reactions are initiated. This is the subject of ongoing investigation.

The time-independent response for decane suggests that KPFM in liquid is possible in ionically-inactive liquids, as was previously reported [39]. In ionically-active liquids, however, the large peak and subsequent exponential relaxation of the cantilever response observed is likely associated with diffuse ion dynamics. To account for these effects, an equivalent circuit approach is adopted, in which the double layer charging is modelled as an idealized capacitor [6] and the ion dynamics can be described in terms of equivalent circuits, where the double layer remains in quasi-equilibrium with the neutral bulk electrolyte. The charging is governed by the transport of ions into the double layer. During the double layer charging, the bulk electrolyte concentration remains nearly uniform, and thus, the bulk electrolyte acts as a resistor in series with the double layer capacitors at each interface. Equivalent circuit models are commonly used in electrochemical measurements, and reportedly describe the electrostatic actuation of microelectromechanical systems [59] and electrostatic force microscopy (EFM) [50] in liquid, analysis of which was performed analytically and verified experimentally to establish the critical actuation frequency such that the timescale is shorter than required for double layer screening.

The associated *RC* timescale can be expressed as

[4]
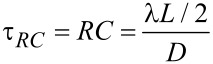


where *D* is the diffusivity of the ions in solution, *L* is the separation of the electrodes and λ is the Debye screening length, given by

[5]
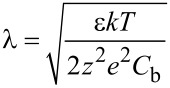


where *k* is Boltzmann’s constant, *C*_b_ is the molar concentration, ε is the dielectric permittivity of the solvent, *T* is the temperature, *e* is the electronic charge and *z* is the ion valence. At low biases (*V*_dc_ < *kT*/*e* ≈ 25 mV) and in the absence of Faradaic reactions, this *RC* time is the relevant timescale of the transient response, e.g., in high-frequency impedance spectroscopy experiments or induced charge electrokinetics, where high-frequency alternating currents are applied [5]. Thus, it would be expected that the *RC* time is the relevant timescale of the transient response in isopropanol or milli-Q water (Figure 2g,j), suggesting a similarity between τ_1_ and τ*_RC_*, as previously reported [38].

Despite the broad applicability of equivalent circuit models in electrochemistry, and the success shown in describing imaging mechanisms in low molarities (≈10 mM) using high frequency (>>MHz) EFM [50], their suitability across all biases (particularly large biases where *V*_dc_ >> 25 mV) and timescales as well as at higher ion concentration is unclear, particularly as the non-uniform evolution of ion concentration (e.g., ion depletion in the neutral bulk electrolyte) cannot be adequately modelled by homogenous circuit elements [37]. Furthermore, the *RC* time is not the only relevant timescale for equilibration of an electrochemical cell, as illustrated by the apparent double exponential decay of 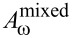 in ionically-active liquids (see Supporting Information File 1, Figure S1 and Figure S2). As previously described [38], the bulk electrolyte diffusion timescale (τ*_L_*) (i.e., the time it takes an ion to diffuse from the bulk electrolyte into the diffuse charge layer), (τ*_L_* = (*L*/2)^2^/*D*), which is much larger than the *RC* time for thin double layers (*L* >> 2λ) becomes increasingly important in the presence of Faradaic reactions or at large applied biases (*V*_dc_ >> 25 mV) for blocking electrodes where the electric double layer adsorbs neutral salt such that the bulk electrolyte becomes depleted [5]. It is likely that τ_2_ is related to ion depletion and subsequent ion diffusion from the bulk electrolyte, suggesting a similarity to τ*_L_* as previously reported [38]. As seen in Figure 2j, more sophisticated descriptions of the electrostatic force than those described in Equations 1–3 are required to account for non-linear effects (e.g., ion crowding and Faradaic reactions) across all bias ranges. Towards a complete understanding of these phenomena, it is expected that the full time-dependent ion transport dynamics, recently developed for ideally polarizable electrodes, will need to be solved numerically for the complex probe–sample geometry [4]. In the presence of Faradaic reactions, this approach must be extended to include the Frumkin correction and other non-linear modifications of the reaction rate associated with diffuse charge [5].

**Figure 3:**
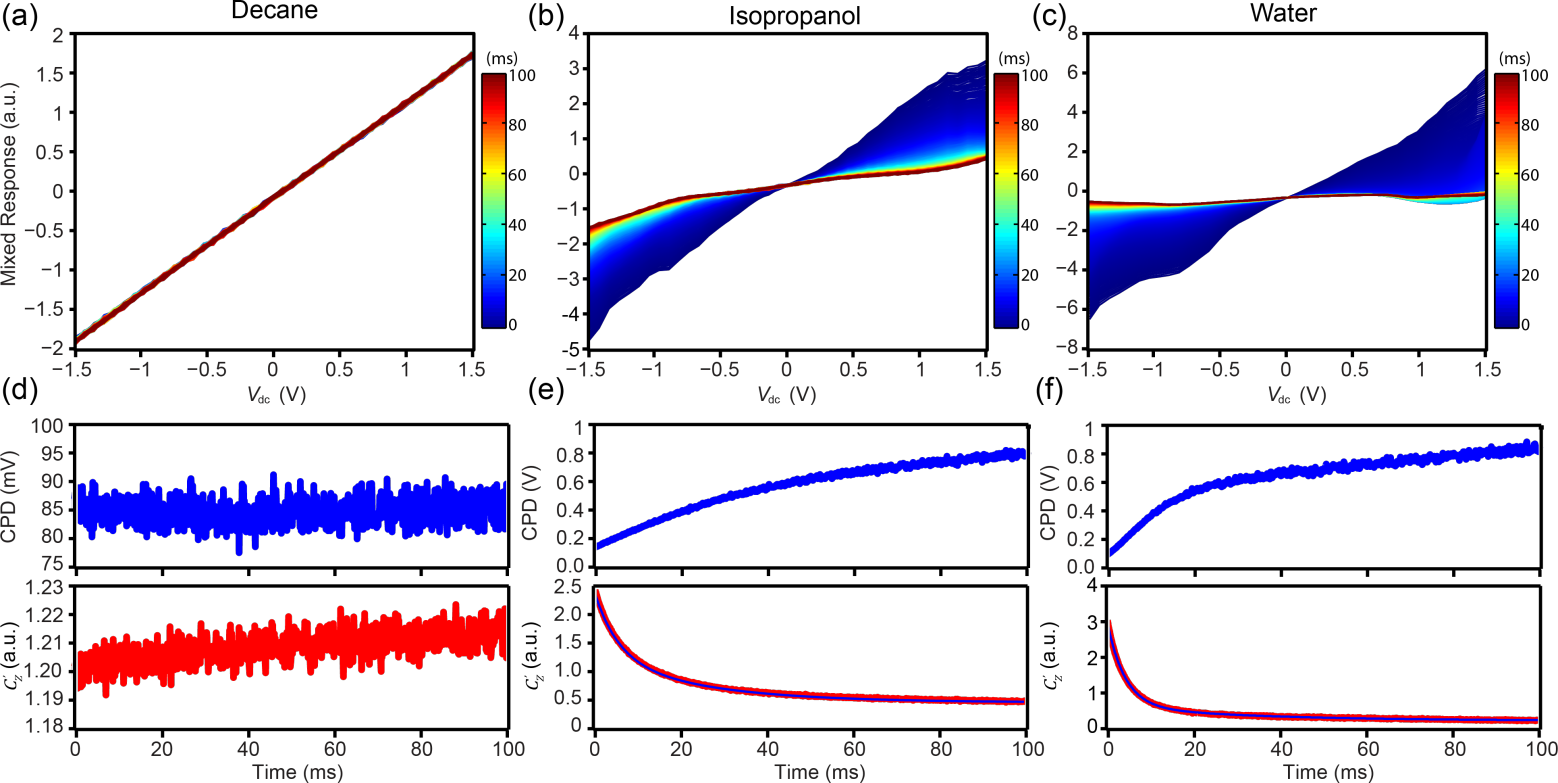
EcFM measurements [bias-on] in (a, d) decane (b, e) isopropanol and (c, f) milli-Q water recorded 200 nm above a grounded Au electrode. (a, b, c) EcFM bias-resolved 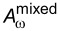 with time represented on the color scale. (d, e, f) The measured CPD and 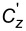 determined from linear fitting of 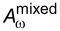 (Supporting Information File 1, Figure S3). Measurements were performed with *V*_ac_ = 0.5 V [25 kHz] applied to the probe.

The presence of a non-linear bias dependence (Figure 1) and time-dependent response (Figure 2) currently precludes the application of KPFM in ionically-active liquids. Figure 3a,b,c show 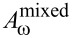 collected in decane, isopropanol and milli-Q water, respectively. Here, 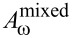 is plotted as a function of *V*_dc_, while the color scale represents the timescale. In decane, 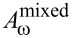 follows a linear bias dependence and no deviation from linearity was observed for all times probed. Under these conditions, EcFM “converges” to KPFM in the sense that EcFM can be used to determine the CPD by linear fitting as is done in OLBS (see Supporting Information File 1, Figure S3) to find the bias at which 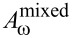 is minimized. The CPD was determined for each time slice as 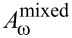 = 0 and the slope of a linear fit was used to measure 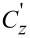 [44]. For decane (Figure 3a,d), both the CPD (85 ± 2 mV) and 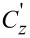 were constant within experimental error with any variations likely to be a result of small changes in probe–sample geometry due to drift in the probe–sample separation during the measurement. These results validate the implementation of KPFM in decane [39]. Since the decane acts like a near-perfect lossless dielectric between probe and sample, the dynamic response is purely capacitive and can be effectively described by Equation 2. The extension of KPFM to operation in ionically-active liquids provides an opportunity to study, e.g., multi-layered charge structures in non-polar electrified interfaces and electrochemical potentials of thin layers and surfactants.

In isopropanol or milli-Q water, when the measurement timescale <<τ*_RC_*, i.e., faster than the time it takes ions in solution to fully screen the charged surface from the probe, a measured value for the CPD can be obtained in a manner similar to that used for decane. In the case of milli-Q water, the bias dependence was fit in a smaller bias window (–500 to +500 mV) to avoid including the observed non-linear behavior at higher biases (see Supporting Information File 1, Figure S2). From the first 2 ms of data the CPD of the Au electrode was found to be 158 ± 8 mV in isopropanol and 112 ± 14 mV in milli-Q water, close to that observed in decane (85 ± 2 mV). The discrepancies between the measured CPD values likely result from physisorption of molecules at the solid–liquid interface [39]. The error is greater for the measured CPD recorded in ionically-active polar solvents than in decane due to gradual changes of the measured CPD in milli-Q water and isopropanol (Figure 3e,f), which varies by >650 mV in both ionically-active polar liquids within the 100 ms measurement as a result of screening by the double layers between probe and sample. This is also reflected in the transient behavior in the capacitance gradient, shown in Figure 3e,f. Again, this transient response was well-described by a double exponent fit (blue line) having relaxation times of τ_1_ = 5.4 ms and τ_2_ = 23.7 ms in isopropanol and τ_1_ = 4.2 ms and τ_2_ = 31.7 ms in milli-Q water. This time dependence in polar liquids, which is absent in decane, prohibits the implementation of KPFM in ionically-active liquids and necessitates the adoption of multidimensional bias- and time-resolved techniques, as demonstrated here.

**Figure 4:**
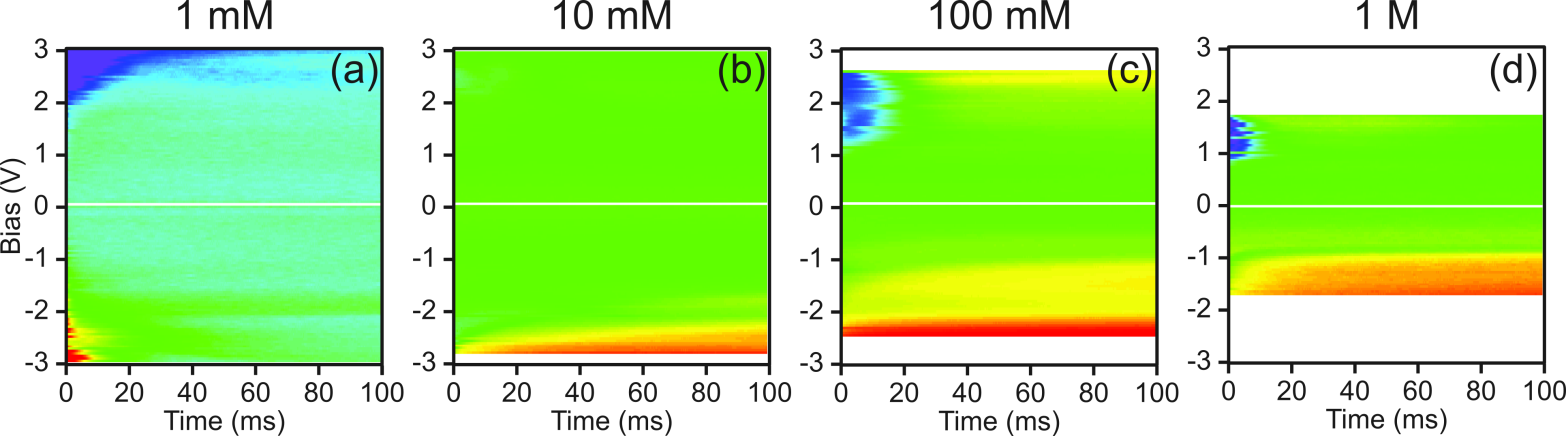
EcFM 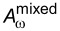 spectra [bias-on] collected 500 nm above HOPG in aqueous solutions of increasing salt concentration (1 mM–1 M NaCl). Measurements were recorded from low to high concentrations using the same probe. Vertical scale ±5 a.u. (data normalized by the mean value at the smallest positive *V*_dc_). Measurements were performed with *V*_ac_ = 1 V [15 kHz] applied to the probe.

Most electrochemical systems of interest (i.e., energy or biological systems), however, require characterization at high ion concentration >100 mM. Thus, to explore the dependence of the ion concentration on the EcFM response, single-point EcFM measurements were performed as a function of NaCl concentration (Figure 4). For measurements performed in the presence of NaCl, the data shows a non-linear bias dependence of 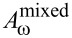. In addition, bubble formation was observed within the applied bias range ≥10 mM, with the threshold for bubble formation being lower for larger salt concentrations. When bubbles became visible, data collection was stopped.

For concentrations ≥10 mM, 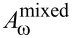 did not fully relax within the measurement time following the application of negative bias. The calculated double layer charging times for Figure 4b, τ*_RC_* = 0.8 µs between tip and sample and τ*_RC_* = 22.8 µs between cantilever and sample (λ = 3.04 nm, *L*_tip_ = 500 nm, *D* = 1 × 10^−9^ m^2^s^−1^ and *L*_cantilever_ = 15 µm), are both well outside the current temporal resolution, which is currently determined by the low pass filter of the lock-in amplifier (100 µs). The slower processes (>>10 ms) are likely a result of irreversible Faradaic reactions.

**Figure 5:**
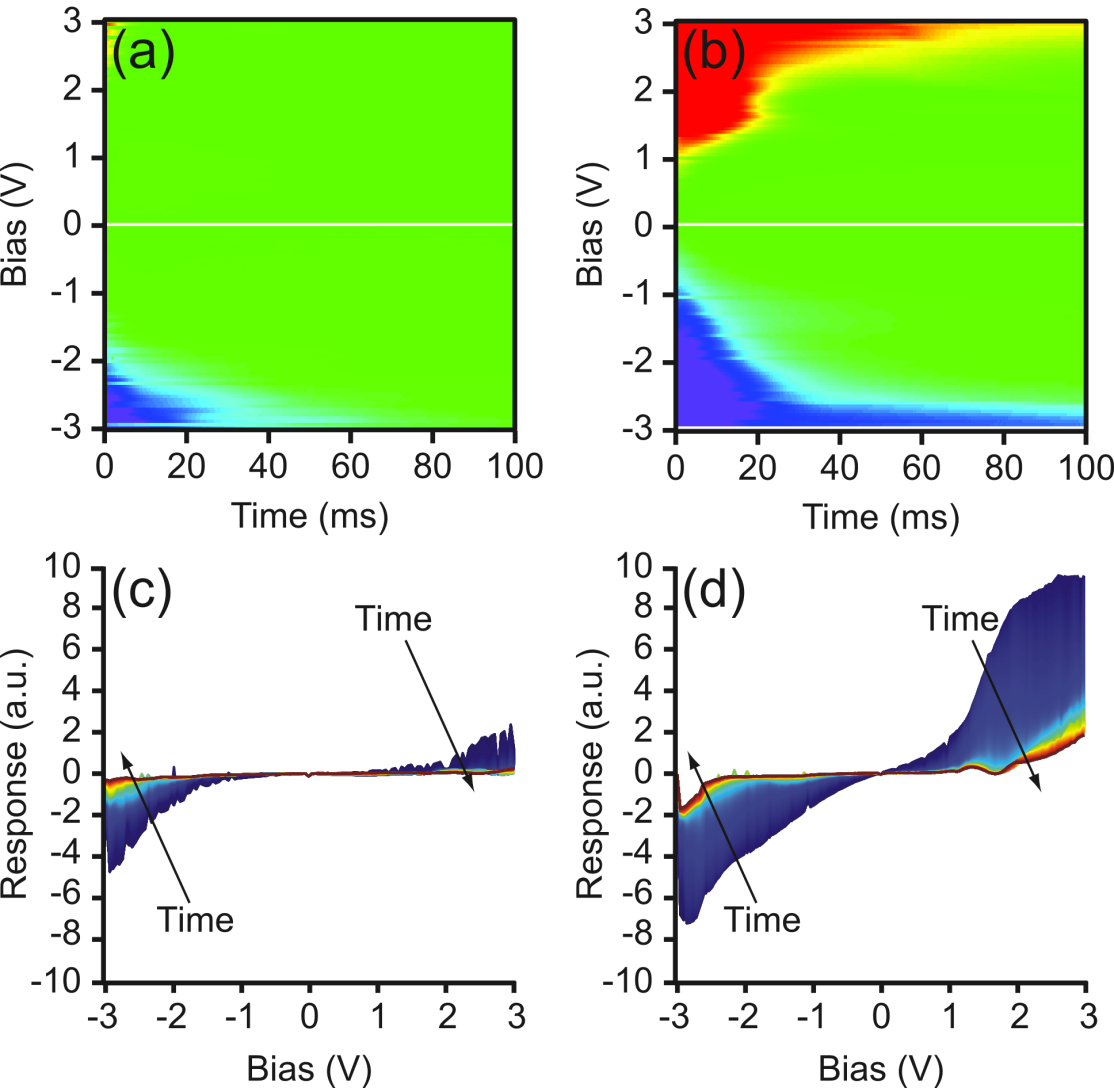
EcFM 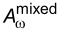 spectra [bias-on] collected 500 nm above (a) HOPG and (b) Au in milli-Q water (vertical color scale ±3 a.u. and 0–4 a.u., respectively). Evolution of 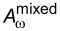 response as a function of time for (c) HOPG and (d) Au surfaces (vertical color scale 100 ms). Measurements were performed with *V*_ac_ = 1 V [15 kHz] applied to the probe.

In Figure 1, HOPG and Au demonstrated different electrochemical response for both OLBS and CV measurements. The differences in electrochemical properties are also visible in single-point EcFM spectra recorded above both surfaces. 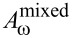 was observed to be non-linear across the full bias range for both Au and HOPG in milli-Q water (Figure 5). The response of 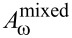 to negative *V*_dc_ for both surfaces were similar in magnitude, however, the response to positive *V*_dc_ was significantly larger for Au than for HOPG. In addition, the signal was less likely to relax within the measurement time for Au than for HOPG. The lower response to positive *V*_dc_ for HOPG as compared to Au can be explained by the inert electrochemical nature of the basal plane of HOPG and the low electrocatalytic activity of HOPG for many redox reactions in comparison to Au, as was previously demonstrated in Figure 1e.

### Mapping local electrochemical reactivity using EcFM

**Figure 6:**
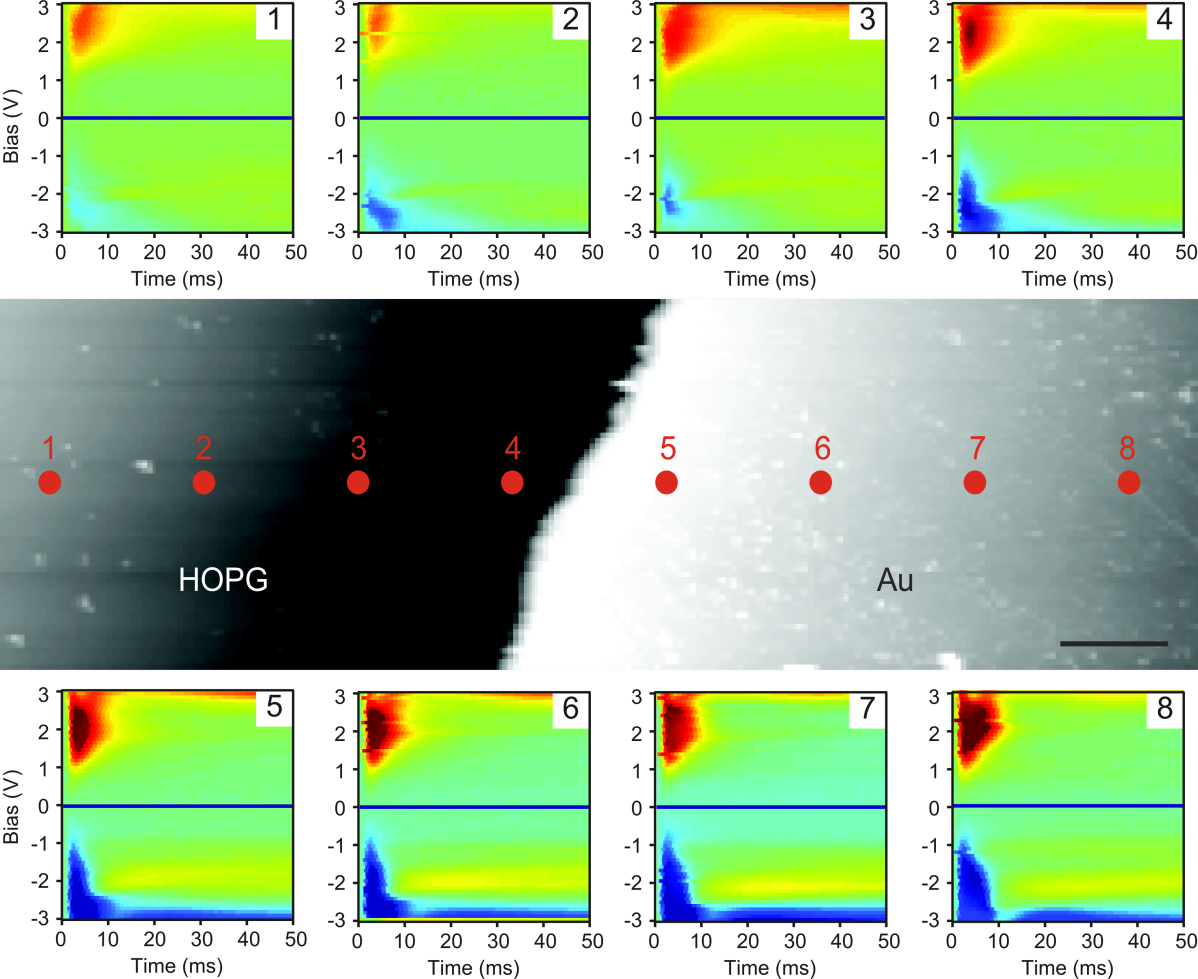
Variability of EcFM 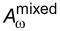 spectra [bias-on] recorded 500 nm above a HOPG/Au boundary in milli-Q water. Topography and markers indicate positions at which the single-point data was collected (scale bar = 5 µm). Vertical color scale ±10 a.u. for inserts 1–8. Measurements were performed with *V*_ac_ = 0.5 V [17.5 kHz] applied to the probe.

Finally, the different electrochemical properties of HOPG and Au are leveraged to assess the spatial variability in the EcFM response for both materials, and thus, the utility of EcFM for spatially-resolved imaging. To show the localization of the EcFM response, a model sample comprising an electrode of Au deposited on a HOPG surface is used. Shown in Figure 6 is an example of EcFM point spectroscopy across a HOPG/Au boundary in milli-Q water. The variability of the spectral data can be seen across the sample surface and a transition close to the boundary of the materials is observed. This variability supports the previously reported conclusion that the data is localized and dependent on the material below the probe, which allows spatially-resolved mapping of electrochemical reactivity using force-based detection [38].

### Statistical analysis of EcFM

The complexity of the electrochemical processes taking place between tip and sample requires the adoption of a multidimensional approach to capture the bias (*V*) and time (*t*) dependence of the response at each spatial (x,y) location on the sample. The corresponding multidimensional data (x,y,*V*,*t*) can become difficult to analyze, particularly if it cannot be reduced by using phenomenological fitting procedures with parameters having a known physical relationship. Indeed, the development of fitting procedures, based on analytical models, is a key requirement for the broad applicability of EcFM across all areas of electrochemistry. However, in the short term, the significant increase in both size and complexity of the data necessitates simple analysis methods capable of dealing with high dimensional information and without relying on a priori physical models. One possible solution to overcome the difficulty in dealing with such high dimensional data sets is by using multivariate statistical approaches. Here, principal component analysis (PCA) [60] is applied to visualize spatial variability within the EcFM data. PCA selects and ranks relevant response components based on variance within the data purely on statistical methods, without employing assumptions regarding underlying physical behavior. In this way, the first eigenvector contains the most statistically relevant information (defined as variance) within the dataset, while the second contains the most statistically relevant information after subtraction of the first, and so on. PCA is used to transform a number of correlated variables into a smaller number of uncorrelated variables called principal components (PCs), where each PC can be represented as an eigenvector and corresponding loading (eigenvalue) map.

**Figure 7:**
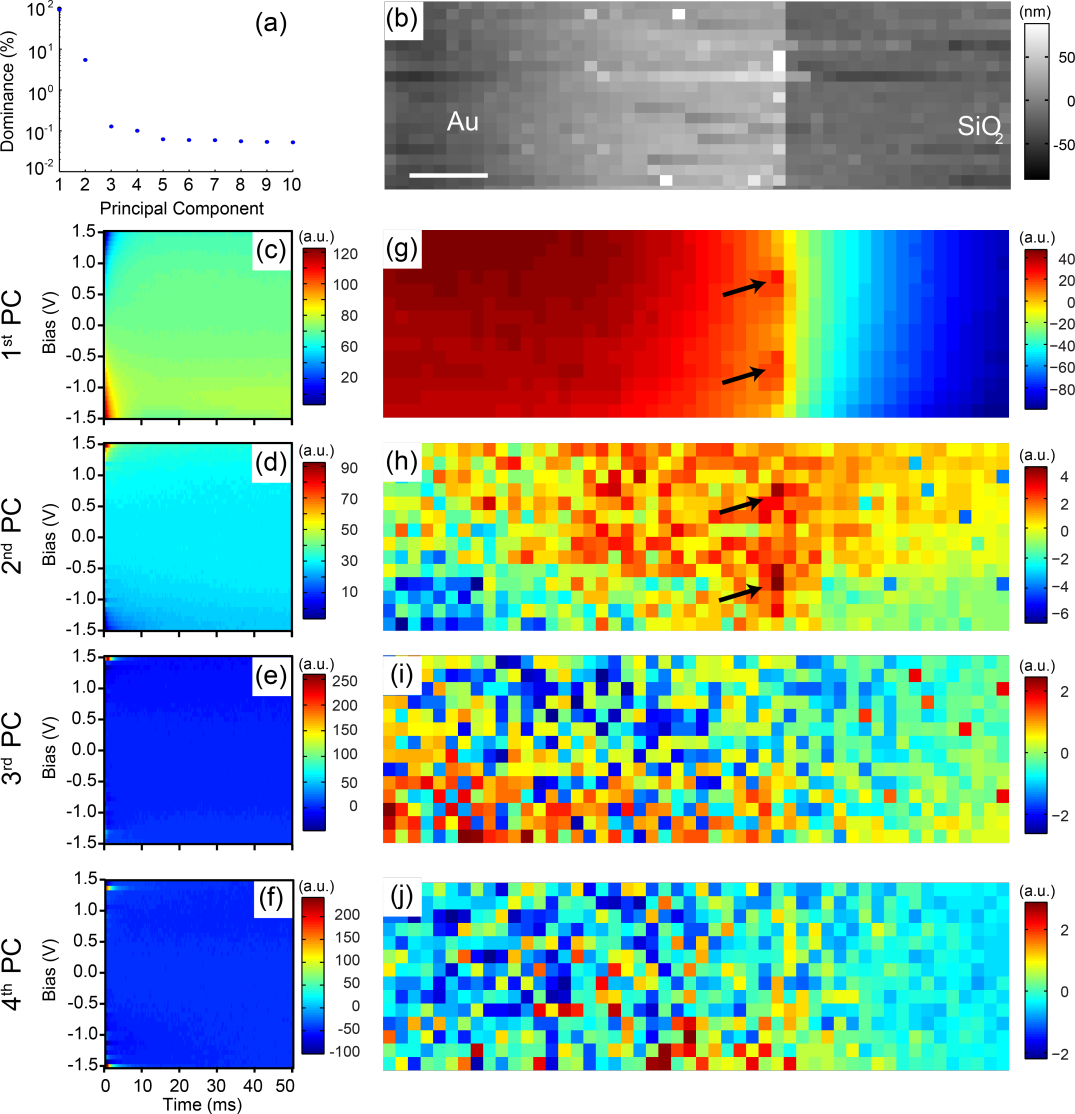
PCA results of a first harmonic EcFM data set on a 50 × 15 grid recorded at a distance of 200 nm above an Au/SiO_2_ boundary (scale bar = 5 µm). (a) Plot of the dominance of the response versus principal component (PC). (b) Reconstructed topography from force measurements during EcFM data acquisition. (c–f) show the first 4 PC eigenvectors respectively and (g–j) showing their corresponding PC spatial maps. Black arrows in the (g) first and (h) second PC spatial maps highlight electrochemical hotspots recognized using PCA.

Figure 7 demonstrates the usefulness of the application of PCA to EcFM data. The EcFM data analyzed was of the first harmonic (bias on) mixed response recorded on a 50 × 15 grid across a metal/insulator boundary. The spatial distribution of the first component demonstrates that PCA separates the overall behavior into metal and insulator regions. The first two PCs contain >97% of the statistically relevant information as shown from the dominance plot in Figure 7a. Loading maps beyond 2nd PC are dominated by noise. Note that within the first two PCs, isolated regions or hot spots demonstrate a different electrochemical behavior from their immediate surroundings. This highlights the usefulness of PCA for dimensionality reduction and qualitative visualization of spatial variations in the EcFM measurements. The corresponding eigenvectors have complicated behavior and do not allow for direct physical interpretation, however, PCA may act as a precursor for more suitable multivariate statistical approaches which do allow physical interpretation (e.g., Bayesian demixing analysis) of the response when combined with analytical modelling [61].

## Conclusion

The feasibility of force-based electrostatic and electrochemical measurements was investigated in liquid by probing the bias- and time-dependent response to a biased probe. The implementation of conventional closed loop-KPFM has been determined to be possible only when the first harmonic response is linear with applied bias and the measurement is performed under equilibrium conditions (i.e., time-invariant response). The operation of KPFM in non-polar liquids was justified by demonstrating a linear bias dependence and a time-invariant relaxation of the first harmonic cantilever response. In the presence of diffuse charge dynamics, however, the linear bias dependence detected at timescales <<τ*_RC_* was found to quickly relax as a result of ion dynamics, precluding the use of KPFM in ionically-active liquids. To overcome this and to further probe bias- and time-dependent ion dynamics and material-dependent electrochemical processes at the probe-sample junction, EcFM was implemented.

Unlike KPFM, EcFM provides a framework to separate different electrochemical processes at the solid–liquid interface based on their bias- and time-dependent response, and thus, has the potential to provide fundamental insights into diffuse charge dynamics. In this work, EcFM has been implemented in ionically-active and -inactive liquids and the usefulness of principal component analysis, which does not require assumptions on physical tip–sample interactions, to map electrochemical behavior across a metal–insulator junction has been demonstrated. It is anticipated that EcFM measurements will be useful in the study of local electro-osmotic flow in microelectrode devices, ion intercalation in capacitor materials, changes in reaction kinetics due to ion adsorption/desorption at the solid–liquid interface and other phenomena central to biological and energy research.

## Experimental

HOPG (Agar Scientific) was cleaved immediately prior to use. Au-coated borosilicate glass substrates (Arrandee) were cleaned in isopropanol and ethanol and rinsed in milli-Q water prior to use. For the Au/HOPG sample, ≈45 nm of Au was deposited on ≈5 nm of Ti by evaporation on top of a freshly cleaved HOPG surface. For the Au/SiO_2_ sample, ≈50 nm of Au was deposited by evaporation on the SiO_2_ surface. During measurements all samples were mounted on a conductive surface using silver paint which was at ground potential with respect to the tip.

All OLBS and EcFM measurements were performed using a commercial AFM system (Asylum Research, MFP-3D) and as-received Pt/Ir-coated (Nanosensors, PPP-EFM) cantilevers with a nominal mechanical resonant frequency and spring constant of 75 kHz and 2.8 N/m, respectively. Linear bias sweeps were performed using a procedure implemented using Igor Pro (Wavemetrics) and the AFM controller, which was used to control the DC bias added to the AC voltage from the lock-in amplifier. For EcFM measurements the tip position was controlled by a custom program written in Igor Pro (Wavemetrics). After positioning the tip in the correct location, the tip was then retracted a predefined distance from the surface and a trigger was used to initiate the electrochemical measurement. In all measurements shown the tip–sample distance was chosen to ensure the interaction was purely long range and that any changes in separation due to drift were small compared to the tip–sample distance. Measurements in Figures 1–3 were performed using a multifrequency lock in amplifier (Zurich Instruments, HF2LI) having a built in electronic adder and Figures 4–7 were performed using two lock-in amplifiers (Stanford research, SR830 and Signal Recovery, 7280) and an external electronic adder (Nanonis, SA4). EcFM measurements were performed with a LabView/MatLab controller implemented on National Instruments PXIe-6124 (Figures 1–3) or 5122/5412 (Figures 4–7) fast AWG and DAQ cards.

Isopropyl alcohol (isopropanol) (≥99.7%, Sigma-Aldrich) and deionized water (milli-Q, Gradient A10, resistance of 18.2 MΩ∙cm) was used for measurements reported in polar solvents. CV traces were collected using a potentiostat (Biologic, SP300) and an Ag/AgCl reference electrode.

## Supporting Information

Double exponent fitting of the transient EcFM mixed response in isopropanol (Figure S1), double exponent fitting of the transient EcFM mixed response in milli-Q water (Figure S2) and linear fitting of the EcFM mixed response for determination of CPD (Figure S3).

File 1 Additional figures.
