# Supplementary material for: Kelvin probe force microscopy in liquid using electrochemical force microscopy
Source: Beilstein J Nanotechnol. 2015 Jan 19;6:201–14. doi: 10.3762/bjnano.6.19 (PMC4311590; doi:10.3762/bjnano.6.19)
Supplement: File 1 — Additional figures. [file Beilstein_J_Nanotechnol-06-201-s001.pdf]

Supporting Information

for

Kelvin probe force microscopy in liquid using electrochemical force microscopy

Liam Collins[1,2], Stephen Jesse[3], Jason I. Kilpatrick[2], Alexander Tselev[3], M. Baris Okatan[3], Sergei V. Kalinin[3,4] and Brian J. Rodriguez*[1,2]

Address: [1]School of Physics, University College Dublin, Belfield, Dublin 4, Ireland, [2]Conway Institute of Biomolecular and Biomedical Research, University College Dublin, Belfield, Dublin 4, Ireland, [3]Center for Nanophase Materials Sciences, Oak Ridge National Laboratory, Oak Ridge, Tennessee 37831, USA and [4]Institute for Functional Imaging of Materials, Oak Ridge National Laboratory, Oak Ridge, Tennessee 37831, USA

Email: Brian J. Rodriguez - Brian.Rodriguez@ucd.ie

* Corresponding author

Additional Figures

Double exponent fitting of the transient EcFM mixed response in isopropanol

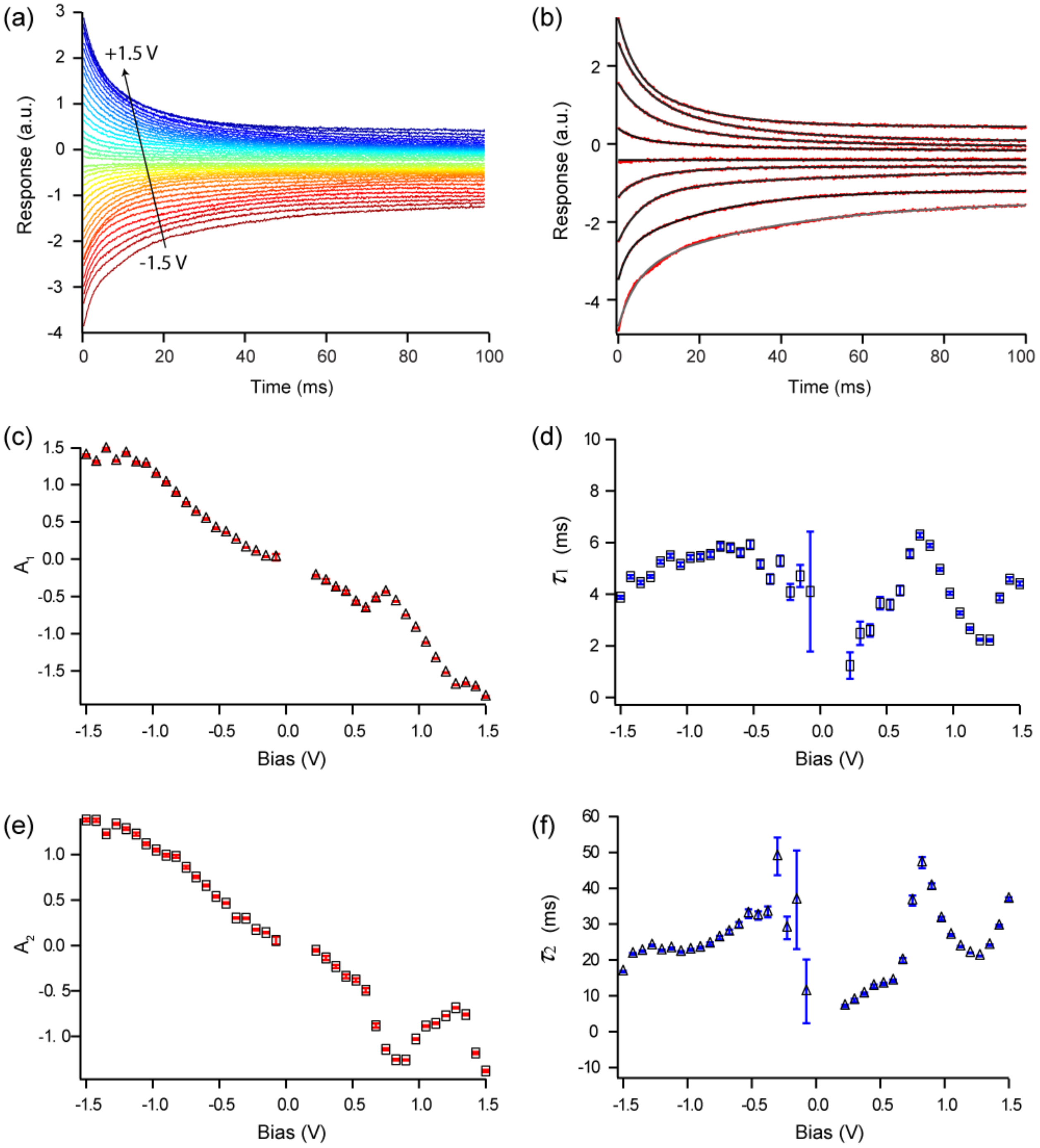

Figure S1: (a) Transient time profiles of EcFM A_{ω}^{mixed} response [bias-on] collected in isopropanol. (b) A selection of double exponential fits (red) to the experimental data (grey) between –1.5 V and + 1.5 V. Bias dependence of the fitting coefficients (± std. dev.) showing (c) A_1 (d) τ_1 (e) A_2 (f) τ_2, where the fitting equation was defined as $f(x) = y_0 + A_1 e^{-t/\tau_1} + A_2 e^{-t/\tau_2}$.

Double exponent fitting of the transient EcFM mixed response in milliQ water

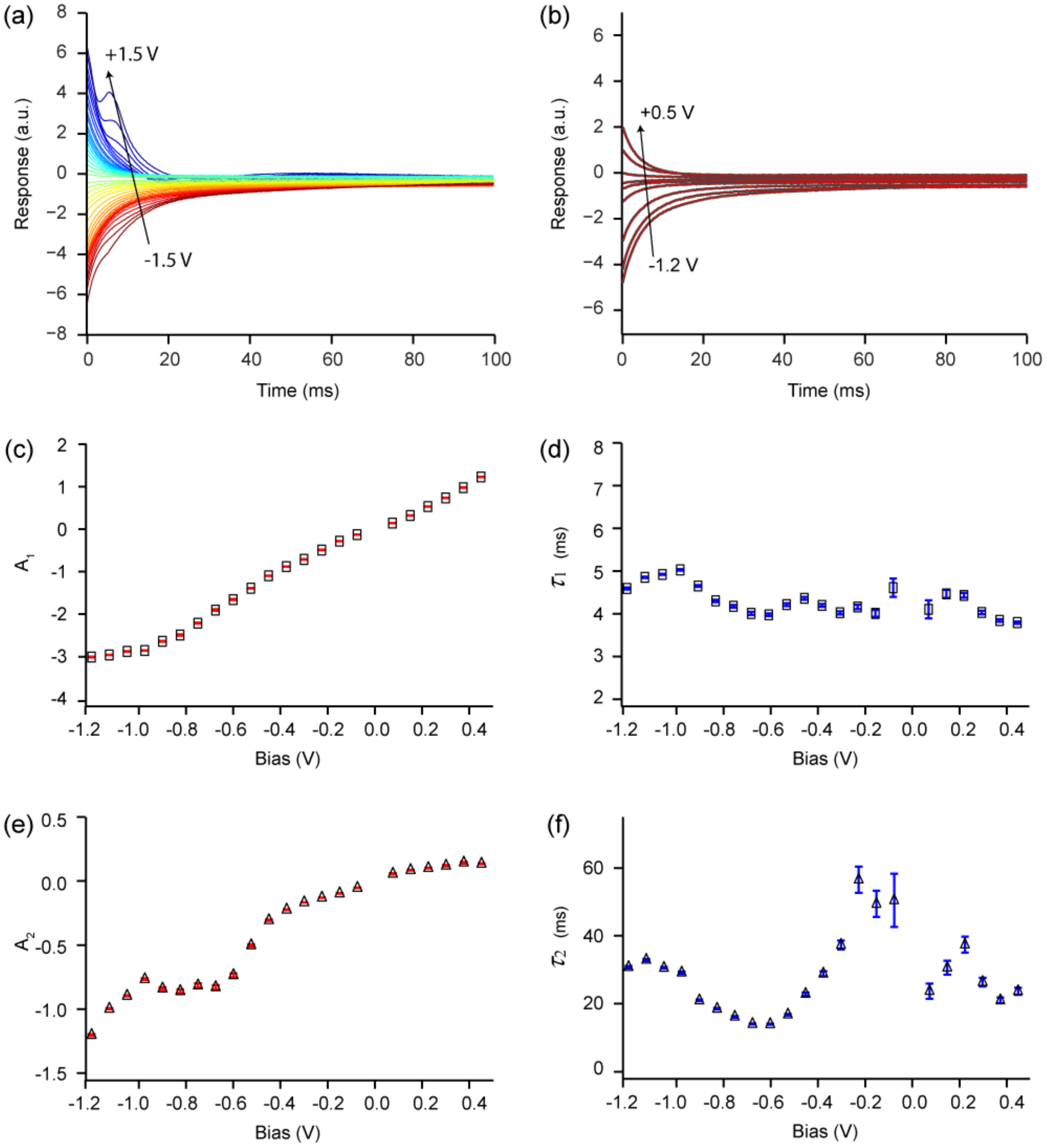

Figure S2: (a) Transient time profiles of EcFM A_{ω}^{mixed} response [bias-on] collected in milliQ water. (b) A selection of double exponential fits (red) to the experimental data (grey) between –1.2 V and +500 mV. Bias dependence of the fitting coefficients (± std. dev.) showing (c) A_1 (d) τ_1 (e) A_2 (f) τ_2, where the fitting equation was defined as $f(x) = y_0 + A_1 e^{-t/\tau_1} + A_2 e^{-t/\tau_2}$.

Linear fitting of the EcFM mixed response for determination of CPD

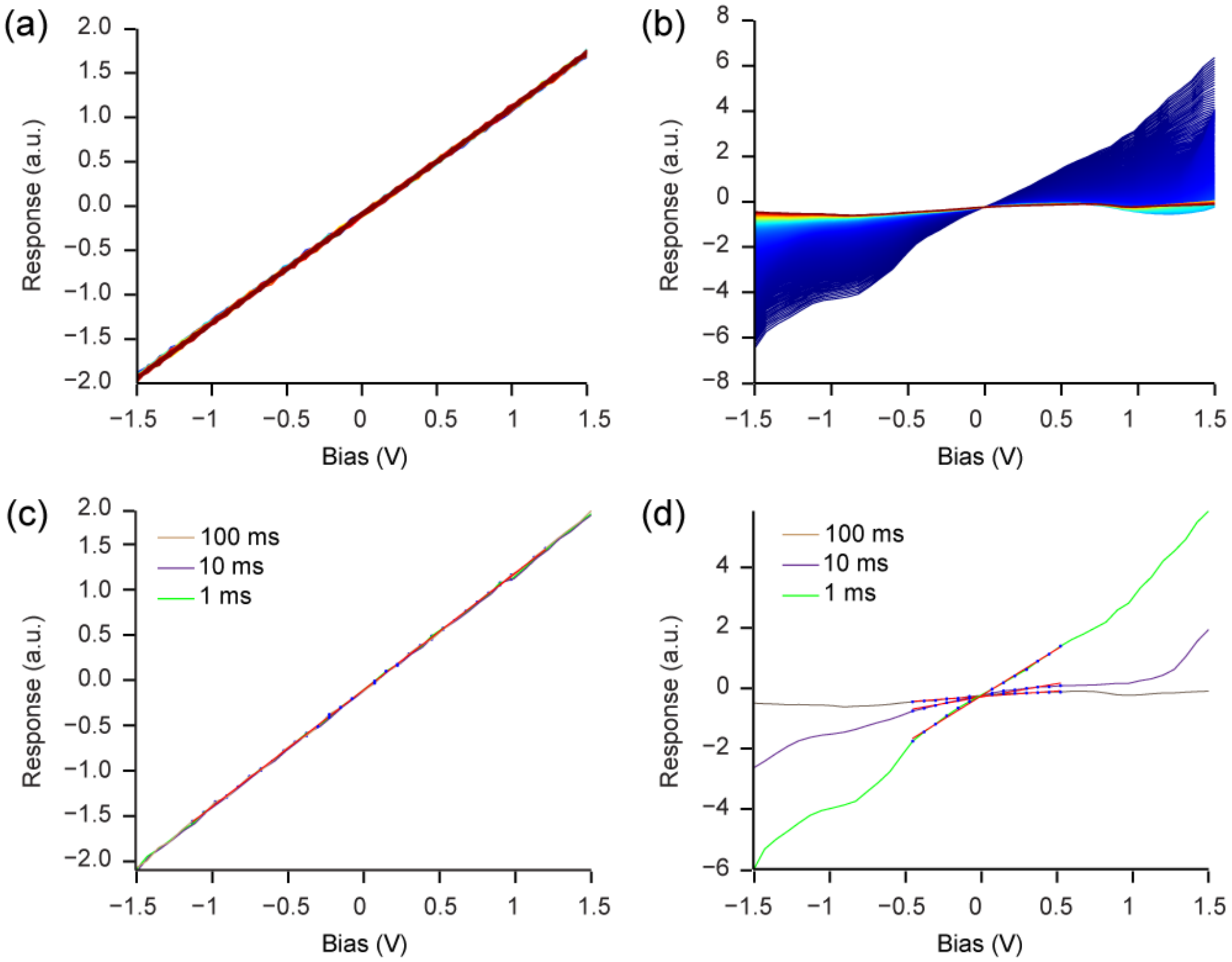

Figure S3: EcFM A_{ω}^{mixed} response [bias on] recorded 200 nm above a grounded Au electrode in (a) decane and (b) milliQ water, as shown in Fig 3. Corresponding linear fitting for 3 timescales (1, 10 and 100 ms) for (c) decane (full bias range) and (d) milliQ water (–500 to +500 mV).
